# Omni-PolyA: a method and tool for accurate recognition of Poly(A) signals in human genomic DNA

**DOI:** 10.1186/s12864-017-4033-7

**Published:** 2017-08-15

**Authors:** Arturo Magana-Mora, Manal Kalkatawi, Vladimir B. Bajic

**Affiliations:** Computational Bioscience Research Center, King Abdullah University of Science and Technology (KAUST), Thuwal, 23955-6900 Saudi Arabia

**Keywords:** Polyadenylation, Prediction, Genomic DNA, Machine learning, Bioinformatics

## Abstract

**Background:**

Polyadenylation is a critical stage of RNA processing during the formation of mature mRNA, and is present in most of the known eukaryote protein-coding transcripts and many long non-coding RNAs. The correct identification of poly(A) signals (PAS) not only helps to elucidate the 3′-end genomic boundaries of a transcribed DNA region and gene regulatory mechanisms but also gives insight into the multiple transcript isoforms resulting from alternative PAS. Although progress has been made in the *in-silico* prediction of genomic signals, the recognition of PAS in DNA genomic sequences remains a challenge.

**Results:**

In this study, we analyzed human genomic DNA sequences for the 12 most common PAS variants. Our analysis has identified a set of features that helps in the recognition of true PAS, which may be involved in the regulation of the polyadenylation process. The proposed features, in combination with a recognition model, resulted in a novel method and tool, Omni-PolyA. Omni-PolyA combines several machine learning techniques such as different classifiers in a tree-like decision structure and genetic algorithms for deriving a robust classification model. We performed a comparison between results obtained by state-of-the-art methods, deep neural networks, and Omni-PolyA. Results show that Omni-PolyA significantly reduced the average classification error rate by 35.37% in the prediction of the 12 considered PAS variants relative to the state-of-the-art results.

**Conclusions:**

The results of our study demonstrate that Omni-PolyA is currently the most accurate model for the prediction of PAS in human and can serve as a useful complement to other PAS recognition methods. Omni-PolyA is publicly available as an online tool accessible at www.cbrc.kaust.edu.sa/omnipolya/.

**Electronic supplementary material:**

The online version of this article (doi:10.1186/s12864-017-4033-7) contains supplementary material, which is available to authorized users.

## Background

**Fig. 1:**
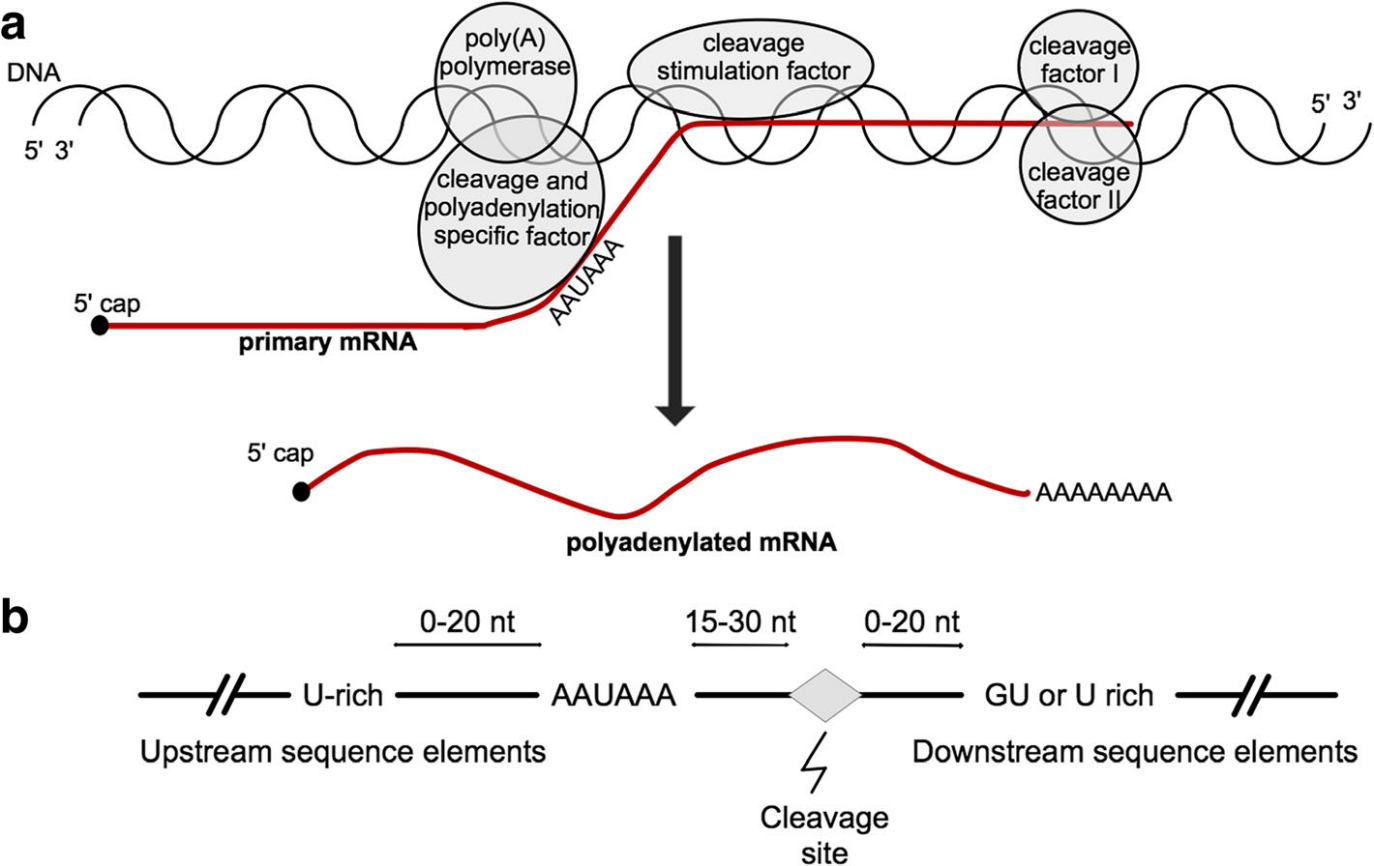
Cleavage/polyadenylation event and general sequence consensus. **a** Diagram representing the proteins involved during the cleavage/polyadenylation event. **b** Schematic representation of the region surrounding the PAS [7]

Polyadenylation is an essential stage of RNA processing during the formation of mature mRNA and occurs in most of the known eukaryotic mRNA sequences [1] as well as in many long non-coding RNAs [2]. The polyadenylation process occurs during RNA processing and involves two stages: 1) cleavage of the primary transcript and 2) the polymerization of an adenosine tail at the downstream of the cleaved mRNA in the case of protein-coding transcripts [3]. The necessary proteins needed for an efficient and accurate cleavage/polyadenylation event include, among others, the cleavage and polyadenylation specific factor, cleavage stimulation factor, cleavage factors I and II, and poly(A) polymerase [4–6] (Fig. 1a). Cleavage and polyadenylation specific factor recognizes and binds to PAS upstream of the cleavage site [7]. Although isolated cleavage and polyadenylation specific factor binds to PAS, the strength of such binding considerably increases when acting along with cleavage stimulation factor [6]. Endonucleic cleavage at the 3′-end is performed by cleavage factors I and II [5]. Refer to reviews [6, 7] for a detailed description of these factors and their interactions.

Although PAS are necessary for the 3′-end polyadenylation, other sequence elements have shown to be necessary for a fully functional cleavage/polyadenylation event, especially the downstream sequence elements, characterized by a GU-rich sequence located up to 20 nucleotides (nt) downstream of the cleavage site [7]. The distance between the PAS and the GU-rich sequence primarily enhances and determines the 3′-end formation [8, 9]. Moreover, upstream sequence elements found upstream of the PAS, are U enriched and often affect the efficiency of the 3′-end formation [8, 10–13]. Finally, the short sequence in the 3′-end of the actual cleavage site may as well have an impact on the efficiency of the process [7, 14] (Fig. 1b). Contrary to the conserved PAS hexamers, downstream sequence elements and upstream sequence elements are highly variable in sequence composition and have not yet been adequately characterized [4]. This sequence variability of the regions flanking PAS causes a major problem in computational prediction of such signals in genomic DNA sequences. Moreover, there is evidence showing that over half of all protein-coding transcripts have alternative PAS [15], resulting in transcripts with variable 3′-end untranslated regions and multiple transcript isoforms [16–19]. Therefore, the correct identification of PAS not only helps in elucidating the 3′-end boundaries of a gene and regulatory mechanisms but also gives an insight into the multiple isoforms resulting from alternative PAS. Furthermore, an accurate predictive model of PAS would help in the identification of PAS for transcripts containing premature termination codons, which are degraded by cellular mechanisms [20].

During the past few decades, several models for the *in-silico* prediction of PAS in genomic DNA and mRNA sequences have been proposed. These models make use of the sequence surrounding the PAS to differentiate true PAS from pseudo-PAS, i.e., hexamers (AATAA, ATTAAA, etc.) that are identical to true PAS hexamers but have no link to the 3′-end polyadenylation. Yada et al. [21] performed a statistical analysis of human genomic sequences surrounding the true PAS covering the region from −80 to +48 where the AATAA hexamer corresponds to positions 0 to 5. From their analysis, the authors observed that C and T/C nucleotides are often found upstream and downstream from the PAS, respectively, and concluded that CAATAAA(T/C) might be regarded as a consensus sequence for PAS. Later, Kondrakhin et al. [22] developed a generalized consensus matrix from a set of 63 vertebrate pre-mRNAs. Elements of the matrix represented the absolute frequencies of nucleotide triplets at each site and were applied to each nucleotide sequence to discriminate PAS from pseudo-PAS. However, when tested on sequences from the adenovirus Ad2 genome, their method produced a high level of false positive predictions. For instance, when their parameters were set to recognize 8 out of 9 true PAS, the model predicted over 1000 pseudo-PAS as true PAS in the Ad2 genome that is of 35,937 nt in length [23]. Subsequently, Salamov and Solovyev [4] developed a model based on a linear discriminant function, from 8 variables defined from a window of 300 nt surrounding the PAS (−100,+200). These variables include, among others, scores from position weight matrices, hexanucleotide composition upstream and downstream, and positional triplet composition. Although the authors achieved better results compared to Yada et al. [21] and Kondrakhin et al. [22], the number of false positives remained relatively high (specificity of ~50%). Tabaska and Zhang [24] developed the polyadq tool consisting of two quadratic discriminant functions (one for each AATAAA and ATTAAA variants) derived from three variables. Their results on two new datasets outperformed the existing methods, especially in the reduction of false positives. In 2003, Legendre and Gautheret [25] developed the ERPIN method based on a probabilistic hidden Markov model. ERPIN used position weight matrices computed for each di-nucleotide in a window of 600 nt surrounding the PAS (−300, +300), and achieved a prediction specificity of 85% for a sensitivity of 56%, resulting in a specificity improvement of 9.7% relative to the polyadq method. Bajic et al. [26] developed the Dragon PolyA tool based on artificial neural networks and self-organized maps for predicting the two most common PAS variants in human (AATAAA and ATTAAA). Their tool improved both sensitivity and specificity by ~5% and 5% on AATAAA variant, respectively, and 11.3% and 7.9% on ATTAAA variant, respectively, relative to those obtained by polyadq. In addition, support vector machine (SVM) approaches have been proposed. Liu et al. [3] derived an SVM model from k-gram and artificially translated amino acid patterns from DNA sequences. Their method includes an entropy-based feature selection process to select the most discriminative features. Their results improved specificity for the three out of four considered datasets compared to ERPIN and polyadq. In 2006, Cheng et al. [27] proposed a polya_svm tool based on an SVM model. Polya_svm is derived from the 15 *cis*-regulatory elements previously found by Hu et al. [28], and achieved an improvement of sensitivity by 33.8% relative to polyadq while preserving the same specificity. In a subsequent study, Xu et al. [29] used SVM-based models for the prediction of PAS in the chromosomal data, i.e., human chromosome 21, and achieved an accuracy of 83%, sensitivity of 90%, specificity of 76%, and a precision of 80%. Akhtar et al. [30] developed the POLYAR tool based on a linear discriminant analysis model. The tool analyzes 600 nt sequences surrounding the PAS and extracts sequence characteristics using position weight matrices, pentamers composition downstream and upstream of PAS, and the distance between *cis-*elements, among others. Moreover, authors divided PAS signals into three categories: 1) PAS-strong, containing the two most common variants AATAAA or ATTAAA, 2) PAS-weak, containing any of the other ten remaining variants and, 3) PAS-less, referring to PAS not having any of the 12 most common variants. For PAS-strong, POLYAR made an improvement of sensitivity by a relative 23% compared to polya_svm, at the expense of reducing specificity by 5.6% relative to polyadq. Both POLYAR and polya_svm obtained similar but considerably lower specificity/sensitivity for PAS-weak and PAS-less sequences, demonstrating the need to characterize signals surrounding other PAS variants. In this direction, Kalkatawi et al. [31, 32] developed the Dragon PolyA Spotter tool (DPS) for PAS prediction in human genomic sequences for each of the 12 most common PAS variants separately. Their method used artificial neural networks and random forest models derived from a set of thermodynamic, compositional and statistical features. DPS method considerably outperformed other results obtained by polyadq, POLYAR and poly_svm tools on the most common PAS variant (AATAAA hexamer). Later, Xie et al. [33] used a hidden Markov model (HMM) to extract latent spectral features from DNA sequences, which were subsequently used as input for a linear SVM model (we refer to this model as HMM_SVM hereafter). The authors considered the same genomic DNA sequences for the 12 PAS variants as used in Kalkatawi et al. [31] and reduced the weighted average error rate by 25% relative to the results generated by the DPS tool. Although considerable progress has been made in the PAS prediction, the predictions still produce an unacceptable level of false positives. Moreover, new biological features surrounding PAS may be defined for the development of more efficient PAS recognition models and may provide a better understanding of the polyadenylation machinery. Furthermore, the application of other machine learning methods may result in more accurate and more robust prediction models.

In this study, we proposed and analyzed a new set of features surrounding the PAS in human genomic DNA sequences and in combination with a prediction model we developed a novel prediction method and tool, Omni-PolyA that predicts PAS in human genomic DNA sequences. Omni-PolyA combines different classification models in a tree-like structure. We implemented a general-purpose optimization technique, namely a parallel genetic algorithm [34] to optimize the Omni-PolyA model structure and its parameters for deriving more accurate results. We compared the performance of Omni-PolyA against results obtained by the methods proposed in Kalkatawi et al. [31] (as implemented in the DPS tool) and Xie et al. [33] (as implemented in the HMM_SVM tool), thereby demonstrating the utility of the proposed feature set and the Omni-PolyA model. Our comparison analysis shows that Omni-PolyA reduces the weighted average error rate by 35.37% relative to the state-of-the-art results [31, 33] for the 12 considered PAS variants.

## Results

The key contributions of our study are the analysis of the genomic sequences for deriving a new set of features capturing elements important for the identification of PAS and the development of a novel method and tool, Omni-PolyA, designed for the prediction of PAS.

### Feature mining from genomic DNA sequences

**Fig. 2:**
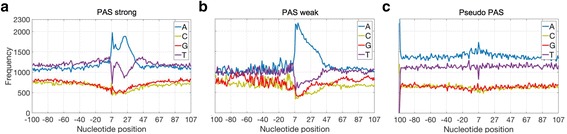
DNA sequence composition. Nucleotide distribution from positions −100 to 100 with respect to a poly(**a**) hexamer. **a** The nucleotide distribution for the genomic DNA sequences in the PAS-strong category. **b** The nucleotide distribution for the genomic DNA in the PAS-weak category. **c** The nucleotide distribution for sequences containing pseudo-PAS. PAS hexamers were removed from the sequence analysis

Tian et al. [15] suggested that polyadenylation events may be determined from the combination of the PAS, DNA elements surrounding PAS, and the binding factors. Clearly, an accurate tool for PAS prediction from genomic DNA sequences would be of great help for real applications, i.e., for finding computationally alternative PAS or as a component of gene finding tools. Therefore, several studies have focused on the identification of *cis*-elements and significant sequence patterns surrounding the PAS [3, 6, 9, 19, 24–28, 31–33]. Notably, Kalkatawi et al. [31] and Xie et al. [33] proposed the most discriminant features and models to date for the prediction of PAS from human genomic sequences. However, selecting the optimal combination of features from a big set and the type of classification model to efficiently utilize them is not trivial. Consequently, we analyzed the genomic DNA sequences flanking the PAS (200 nt long sequences compiled by Kalkatawi et al. [31], see Methods) to obtain new biologically significant features. For better understanding the differences and variations between PAS, we followed the PAS categorization proposed by Akhtar et al. [30]. As such, PAS are divided into two categories: 1) PAS-strong sequences containing AATAAA or ATTAAA variants, and 2) PAS-weak sequences with any of the ten other considered PAS variants. Fig. 2a and b show the different sequence composition between PAS-strong and PAS-weak signal surroundings, respectively. In general, PAS-weak variants show a notably higher enrichment of adenine downstream of PAS, while the upstream region presents greater variability of nucleotide composition compared to PAS-strong. Interestingly, the enrichment of nucleotides A/T downstream of pseudo-PAS (Fig. 2c) may suggest similarities to the functional PAS. Consistent with previous studies [15, 19] this may indicate that there may be functional PAS within coding DNA sequences. However, this hypothesis would have to be tested in a laboratory. It is important to note that Fig. 2 shows the averaged DNA distribution for all sequences. Consequently, not all sequences necessarily contain the same characteristics as observed in the figure. Nonetheless, these observations reveal the main differences between PAS-weak and PAS-strong sequences, which can be used to generate a new set of discriminant features. Based on this analysis, we propose a feature set represented by 218 numeric values (referred to as Omni-PolyA feature set). The Omni-PolyA feature set includes mono-nucleotide and di-nucleotides frequencies in particular regions of the genomic DNA sequences, i.e., downstream, upstream, and in-frame codons with respect to the PAS hexamer, among others. Novel and more specific features determined, make use of the entropy and positional information gain of the nucleotide content to determine the most relevant sequence positions. Moreover, we calculate a sequence score derived from 2-mer weight matrices, which capture the di-nucleotide characteristics for the entire sequence surrounding the PAS. Additional file 1: Table S1 shows the list of the 218 compiled features in Omni-polyA feature set.

### Omni-PolyA method

**Fig. 3:**
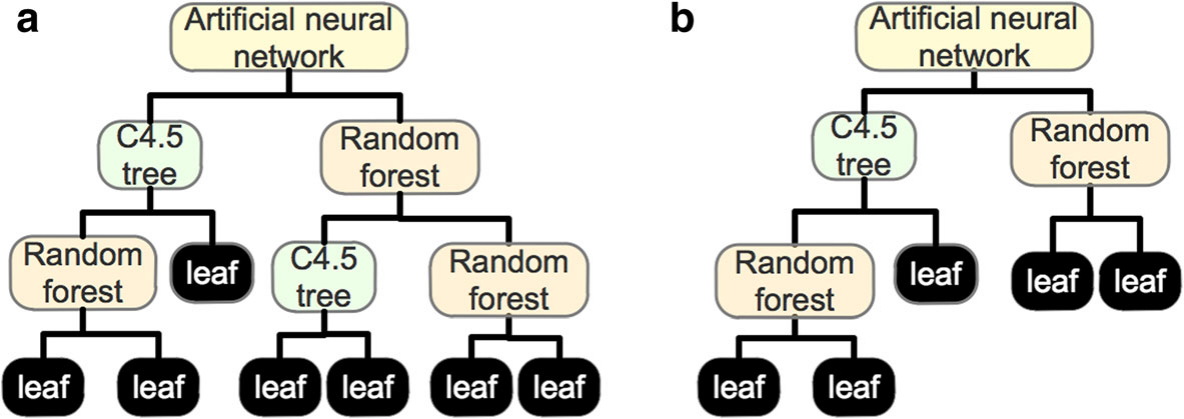
Omnivariate decision tree. **a** Illustration of the omnivariate decision tree structure where each non-terminal node may be an artificial neural network, random forest, multinomial logistic regression or a C4.5 model. **b** An example of the simplified tree resulting from the genetic algorithm optimization

In contrast to typical univariate decision trees (where decision splits are usually made from a single feature threshold), omnivariate decision trees allow the tree to use a more complex classification model at each non-terminal node [35]. Each non-terminal node in the omnivariate decision tree may use a different classification model depending on the data reaching the node. Therefore, this procedure offers the advantages of multiple classification models to learn from different subsets of data. While the method is generic, in our implementation we considered the following classification models for non-terminal nodes: 1) C4.5 univariate decision tree, 2) artificial neural network, 3) random forest and 4) multinomial logistic regression model (Fig. 3a). However, the key challenges rely on determining the depth of the tree and the classification models used at each non-terminal node [36–38]. For this, we implemented a genetic algorithm for optimizing the following processes: 1) tree pruning, where non-terminal nodes with little or no contribution to the classification performance are deleted, and 2) classification model selection and parameters tuning for each non-terminal node. Figure 3b shows an illustration of the resulting tree after the genetic algorithm optimization.

### Comparison of performances in PAS prediction

Results reported in this section were obtained from a 5-fold cross-validation in agreement with benchmark results published for DPS and HMM_SVM state-of-the-art tools for poly(A) signal prediction (see Methods). Table 1 shows the prediction error rates achieved by deep neural networks (DNN), Omni-PolyA and, DPS [31, 32]. It is important to note that DPS, DNN, and Omni-PolyA models were derived by using the same 274 features proposed by Kalkatawi et al. [31] and we refer to this set as DPS feature set. Results in Table 1 show that the DPS feature set may be used to derive more accurate classification models than those reported by Kalkatawi et al. [31, 32]. For example, DNNs consist of a multi-layered architecture that transforms the data representation at one level to a higher and more abstract level [39]. Such an approach has shown to outperform different models applied to solve some challenging tasks in the fields of bioinformatics and cheminformatics [39–41]. Although DNNs typically require a considerably large training set in order to tune a network properly, DNN reduced the weighted average error rate even for the less common PAS variants and reduced the weighted average of the error rate by 11.32% compared to DPS that used the same feature set. These results demonstrate that different models may be better suited for each of the PAS variants. Furthermore, the Omni-PolyA model derived by using the DPS feature set, reduced the weighted average of the error rate by 26.85% and 33.60%, compared to DPS and HMM_SVM, respectively (Additional file 2: Table S2 shows the results obtained by DPS, DNN and Omni-PolyA using the DPS feature and HMM_SVM). We did not consider the latent spectral features proposed by Xie et al. [33] used to derive HMM_SVM due to the high number of features (~10,000) that would result in an unacceptable compute time for non-linear models used as decision nodes in our Omni-PolyA setup based on genetic algorithms. Therefore, we analyzed the genomic sequences and proposed the Omni-PolyA feature set (see the Feature mining from genomic DNA sequences subsection). To assess the discrimination capabilities of the Omni-PolyA feature set, we used these features to derive an Omni-PolyA model for each PAS variant. Results in Table 1 show that the classification error rate, reduced for 8 of the 12 variants with an average improvement of 7.74%, relative to Omni-PolyA derived by using the existing DPS feature set (fifth and sixth columns, Table 1). Overall, this represents a relative error rate improvement of 32.51% and 38.74% over DPS and HMM_SVM state-of-the-art tools, respectively (Additional file 3: Table S3 shows the false positive and false negative rates). Nevertheless, Omni-PolyA achieved an inferior performance on some of the variants from the PAS-weak category compared to HMM_SVM. This may be due to the limited amount of training data for variants with only few hundred sequences. In general, overfitting the training and validation data, outliers, and noise, are among the most relevant problems when deriving classification models from small datasets. For example, in the less frequent PAS variants, the Omni-PolyA algorithm would train a model using a very limited number of sequences, which may derive an unstable and inaccurate classifier. This data limitation is known to considerably affect the performance of the classification models [42, 43]. The creation of synthetic samples, data processing (i.e., noise removal, feature selection, etc.), and data pooling (from similar sub-problems) are among the possible solutions to address the small data problems in deriving more stable classifiers. Notably, the straightforward possibility for deriving robust models for the less frequent PAS variants is to pool the data from different PAS variants with similar nucleotide distributions. Additional file 4: Figure S1 shows the nucleotide distribution for the true PAS sequences for the PAS-weak variants in which we observe a consistent nucleotide enrichment pattern in some sequence regions (i.e., enrichment of T nucleotide in the downstream region 25–50 and the overall A and T enrichment in the 200 nt flanking the PAS). Therefore, we expanded the training data by pooling the PAS-weak variants. For instance, to derive a model for AATAGA variant, the data from the nine remaining PAS-weak variants and the respective training portion from AATAGA variant are used for model tuning (see Methods). Table 1 (seventh column) shows that by pooling the PAS-weak variants, the error rate reduced for eight out of the 10 PAS-weak variants. Considering the best performing Omni-PolyA model for each variant, the classification error rate reduced by 35.37% and 41.34% relative to DPS and HMM_SVM, respectively (Table 2). Notably, the largest error reduction was obtained for the two most common PAS variants (PAS-strong), representing an improvement of 36.95% and 49.50% relative to results achieved by the DPS and HMM_SVM tools.

**Table 1:** Error rate comparison between DPS, DNN and Omni-PolyA derived by using different feature sets from benchmark poly(A) dataset

Variant	Size	Error rate (%)				
		DPS model	DNN model	Omni-PolyA model		
		DPS feature set	DPS feature set	DPS feature set	Omni-PolyA feature set	Omni-PolyA feature set PAS-weak data pooled
AATAAA	5190	23.72	16.80	**14.02**	14.20	14.20
ATTAAA	2400	16.63	15.50	14.00	**12.50**	**12.50**
AAGAAA	1250	14.00	16.88	11.84	**10.80**	11.36
AAAAAG	1230	8.05	8.29	**4.87**	5.85	5.45
AATACA	880	20.00	17.72	**13.52**	14.09	**13.52**
TATAAA	780	18.08	21.28	20.38	14.74	**13.85**
ACTAAA	690	23.33	23.04	19.56	16.23	**14.49**
AGTAAA	670	19.55	22.98	16.71	14.77	**13.13**
GATAAA	460	21.74	16.73	13.69	10.65	**8.48**
AATATA	410	18.05	20.00	16.82	15.85	**13.41**
CATAAA	410	20.00	26.34	24.14	**14.39**	**14.39**
AATAGA	370	18.38	15.40	12.93	12.97	**11.62**
Average		19.25	17.07	14.08	12.99	**12.50**

*‘Size’* corresponds to the number of samples for each PAS motif variant. The *‘error rate’* is the percentage of misclassified motifs; it is equal to 1-accuracy. DPS results correspond to those obtained by applying the method described in Kalkatawi et al. [31]. ‘*Average’* denotes the weighted average of a column. The error rate of the best performing model for each PAS variant is highlighted in bold. Columns 5–7 show the results obtained by Omni-PolyA derived from different feature sets. Seventh column results are obtained by pooling the PAS-weak variants sequences to expand the training data (see Methods)

**Table 2:** Error rate comparison between best performing Omni-PolyA model and state-of-the-art results in benchmark poly(A) dataset from [31]

Variants	Size	Error rate (%)		
		DPS	HMM_SVM	Omni-PolyA
AATAAA	5190	23.72	28.13	*14.02*
ATTAAA	2400	16.63	23.96	*12.5*
AAGAAA	1250	14.00	10.96	*10.8*
AAAAAG	1230	8.05	8.62	*4.87*
AATACA	880	20.00	19.89	*13.52*
TATAAA	780	18.08	16.79	*13.85*
ACTAAA	690	23.33	26.38	*14.49*
AGTAAA	670	19.55	23.13	*13.13*
GATAAA	460	21.74	12.83	*8.48*
AATATA	410	18.05	14.15	*13.41*
CATAAA	410	20.00	*14.15*	14.39
AATAGA	370	18.38	*8.11*	11.62
Average		19.25	21.21	*12.43*

DPS and HMM_SVM results correspond to those obtained by the methods described in Kalkatawi et al. [31] and Xie et al. [33], respectively. One observes that the relative decrease of the weighted average error rate of Omni-PolyA compared to DPS and HMM_SVM is 35.37% and 41.34%, respectively. The error rate of the best performing model for each PAS variant is italicized

The results discussed in Table 1 and Table 2 are obtained by using the data collected by Kalkatawi et al. [31], which has been used as a benchmark in subsequent studies. However, to account for the current annotation for GRCh37, we used the GENCODE Poly(A) feature annotation (release 19) [44] to extract the true PAS sequences (see Methods). Consistent with the previously discussed results, Table 3 shows that Omni-PolyA considerably outperformed the state-of-the-art methods, reducing the error rate by 6.86% and 20.63% relative to the results achieved by DPS and HMM_SVM tools, respectively. Notably, the largest reduction in the error rate was observed in the PAS-weak variants, in which data were pooled to increase the volume of the training data. For PAS-weak, Omni-PolyA reduced the weighted average of the error rate by 11.50% and 32.64% relative to DPS and HMM_SVM tools, respectively. We want to highlight that when the error rates of a predictive system are less than 20%, it appears to be significantly harder to reduce the error further.

**Table 3:** Error rate comparison between Omni-PolyA and state-of-the-art methods in GENCODE poly(A) data

Variant	Size	Error rate (%)		
		DPS	HMM_SVM	Omni-PolyA
AATAAA	24,310	25.49	27.91	23.96
ATTAAA	7098	25.59	33.48	24.20
TATAAA	1640	26.52	36.83	25.86
AGTAAA	1306	26.67	34.77	23.07
CATAAA	682	30.88	38.38	26.91
AATATA	634	24.41	36.98	22.06
GATAAA	528	28.11	37.31	23.26
AATACA	368	32.97	33.89	24.72
AAAAAG	342	31.18	41.76	29.41
ACTAAA	314	28.89	39.03	24.51
AAGAAA	250	31.60	36.00	26.80
AATAGA	100	34.00	40.00	23.00
Average		25.93	30.43	24.15

One observes that the relative decrease of the weighted average error rate of Omni-PolyA compared to DPS and HMM_SVM is 6.86% and 20.63%, respectively

## Discussion

### Survey of PAS-associated genomic features

To capture the sequence variations (Fig. 2a-c) and essential elements involved in the polyadenylation event, we proposed Omni-PolyA feature set containing 218 numeric features (see Additional file 1: Table S1). This set includes features such as positional information gain, scored derived from 2-mer weight matrices, numerical DNA structural profiles, among others. Here, we present a brief survey of the most discriminative features and their biological interpretation.

#### Positional information gain

**Fig. 4:**
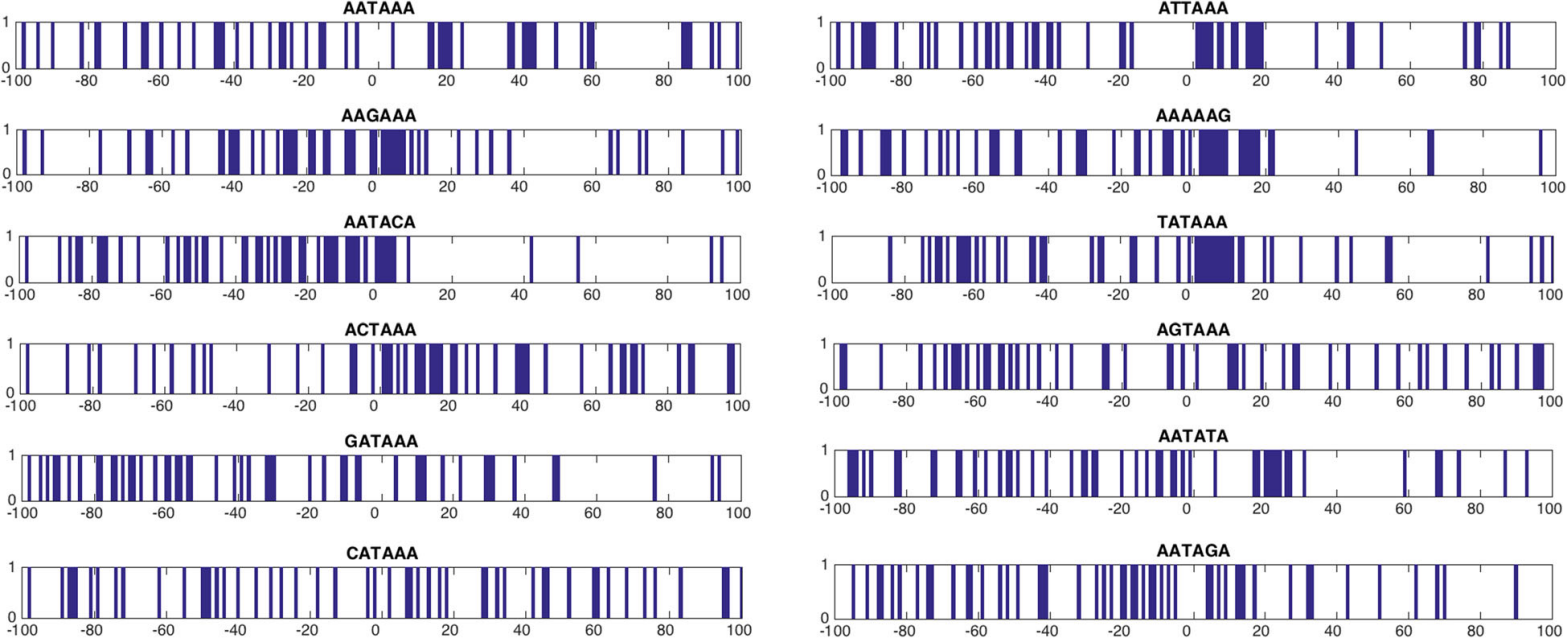
Positional information gain. Blue bars represent the 50 most discriminative positions for each variant

It is clear that PAS-weak/strong and pseudo-PAS sequences, on average, differ in sequence composition, most notably in the region (−20, 40) surrounding PAS (Fig. 2a-c). However, we asked if other regions and independent positions may also be relevant for the polyadenylation event. Therefore, to accurately detect the most discriminative positions in a systematic manner, we calculated the information gain independently for each position of the DNA sequence surrounding PAS (see Methods). Consequently, positional information gain detects the positions within the sequence with the highest contribution for differentiating PAS from pseudo-PAS. In agreement with the PAS consensus shown in Fig. 1, positional information gain identified regions in the proximity of PAS for most of the variants (Fig. 4), where proteins involved in the mRNA cleavage/polyadenylation are expected to bind. Interestingly, the AATACA variant revealed the importance of the upstream region, showing that the downstream segment does not contain significant differences with respect to the pseudo-PAS sequences. Finally, the least common variants, e.g., AATAGA, CATAAA, and AGTAAA, show that the relevant discriminatory positions are spread over the 200 nt sequence and show no apparent significant segment. However, a non-linear classification model may be able to use the relevant positions for classification of PAS for each of the variants. As such, we considered the positional information gain to 1) calculate an overall sequence score, and 2) compute the nucleotide frequency for the top ranked positions based on the information gain (see Methods).

#### Numerical structural profile and PAS

**Fig. 5:**
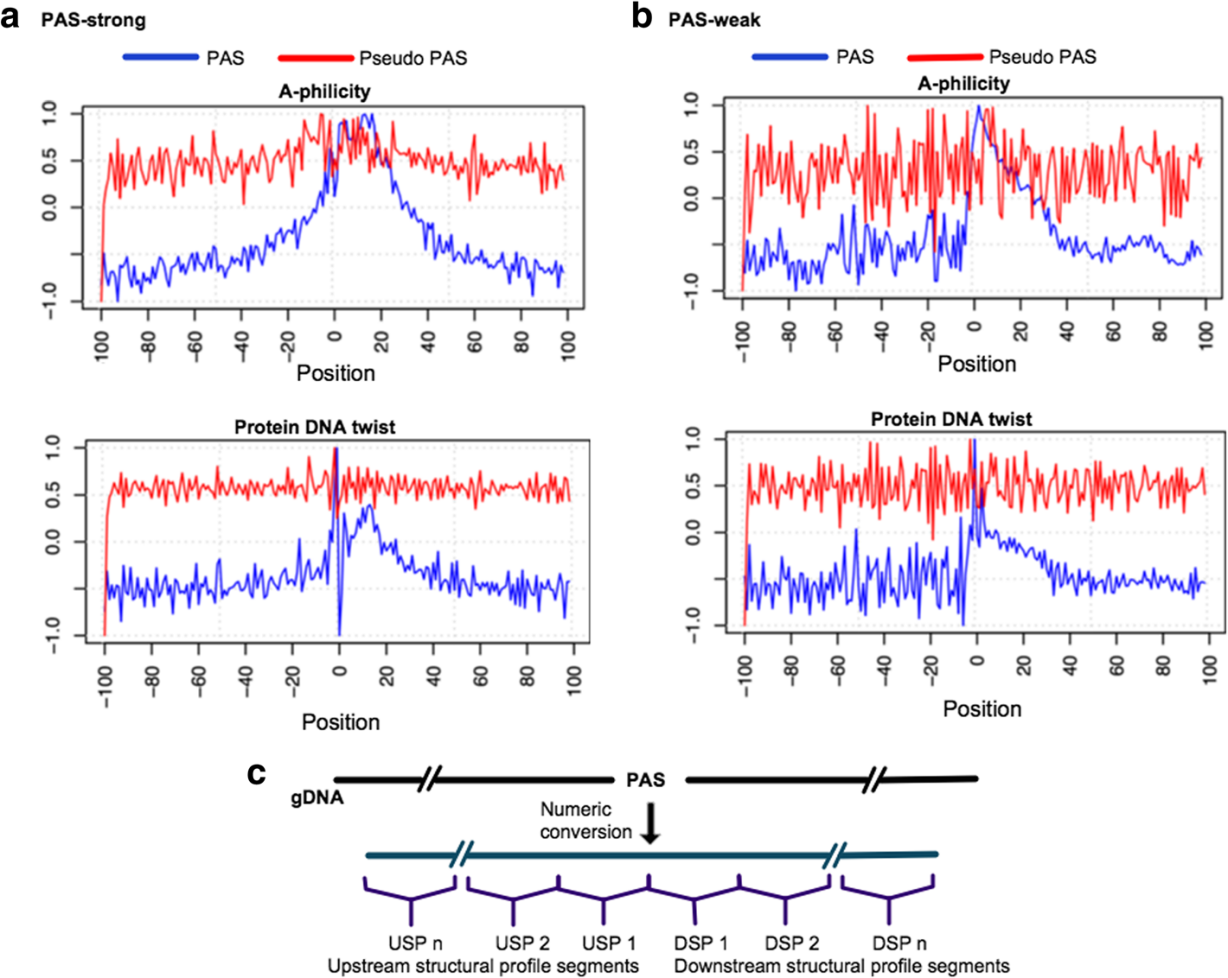
Sequence structural profiles. **a** A-philicity and protein DNA twist numerical representation of the genomic sequences around the PAS-strong variants (sequences of 200 nt in length). Numerical representations show the actual average values over all sequences for each position. **b** Similarly to (**a**), this shows A-philicity and protein DNA twist numerical representations of the genomic sequences for the PAS-weak variants. **c** The short segments used to calculate a granular structural profile

DNA sequences may be converted into a numerical representation to characterize different physical and chemical interactions, i.e., double helix organization, predisposition to interact with other proteins, etc. [45]. These structural profiles have been used in the literature for characterizing genomic signals, i.e., promoter regions [46, 47] and PAS [31, 32]. In total, we used 16 different DNA numeric conversions (conversion tables were obtained from [46]) to define the 200 nt sequences flanking PAS. Notably, A-philicity [48] and protein DNA twist [45] profiles (Fig. 5a-b) show a clear distinguishing pattern for both PAS-strong and PAS-weak compared to pseudo-PAS. A-philicity profile represents the propensity of the DNA to form an A-DNA double helix. Recently, DiMaio et al. [49] suggested that protein binding causes the DNA to adopt an A-form. Similarly, a protein DNA twist profile indicates that both PAS-strong/weak sequences are likely to be deformed by proteins only when within the proximity of PAS (peak around PAS in Fig. 5). Although these structural profiles show a similar pattern, they are, in fact, capturing different information of the sequences and, both profiles accurately detect the region where cleavage and polyadenylation specific factor, cleavage stimulation factor, cleavage factors and poly(A) polymerase proteins are expected to bind. Interestingly, these profiles suggest that the upstream segment in PAS-weak variants is more irregular as opposed to PAS-strong variants. Other numerical conversions were also considered to describe the sequence surrounding PAS. Namely, propeller twist [50], bendability [51], duplex stability free energy [52], DNA bending stiffness [53], stability energy of Z-DNA [54], DNA denaturation [55, 56], nucleosome position preference [57], and base stacking energy [58]. However, B-DNA twist [59], and duplex stability disrupt energy [60] were the least contributing numerical conversions (Additional file 5: Figure S2 and Additional file 6: Figure S3 show the 16 considered structural profiles for PAS-strong and PAS-weak, respectively).

The next key factor is to determine how to use the information from different structural profiles for capturing relevant information. One option is to consider each numerical position in the sequence as independent input for a classification model (representing 199 features for a di-nucleotide structural profile around the 200 nt sequence). As such, we asked if A-philicity numerical representation alone could be used to identify PAS correctly. However, Omni-PolyA achieved ~24.5% error rate for the prediction of PAS-strong variants (compared to 14% when the DPS feature set is used, see Table 1). Although A-philicity numerical representation can moderately discriminate PAS from pseudo-PAS, a combination of several numerical profiles may grant better discrimination capabilities. However, considering the 199 numeric values for each numerical representation would result in a considerably large feature set (~3100 from the 16 numerical representations). The high number of features would lead to complex models trained to use many irrelevant features (assuming that not all positions contribute to the correct classification of PAS). For this, Kalkatawi et al. [31, 32] combined all di/tri-nucleotide representations from each structural profile into a single score, resulting in one feature per structural profile. Arguably, representing all numeric representations in a sequence by a single score may incur a loss of information. For instance, the discriminant information in the region surrounding the PAS (−20, 40) may be diluted by information from less relevant regions. Therefore, we divided the sequence into sub-sequences of 25 nt and calculated the average of each of these (Fig. 5c), resulting in 8 features for each numerical representation. This procedure not only reduces the number of features but also captures different downstream/upstream elements while minimizing the noise of the independent positions.

## Conclusions

In summary, this study shows a comparison of various tools and models applied to the prediction of the 12 most common PAS variants in human genomic DNA sequences. Moreover, by analyzing the differences between PAS-strong, PAS-weak, and pseudo-PAS sequences, we have identified a set of relevant features that may be involved in the regulation of the polyadenylation machinery. In agreement with the consensus of the mammalian PAS (Fig. 1), positional information gain identified relevant regions in the proximity of most of the PAS variants (in the seven most common PAS variants). Conversely, positional information gain showed no clear segments in the rest of the less common variants, possibly indicating the weaker presence of *cis*-regulatory elements in such variants. Interestingly, the AATACA variant revealed the importance of the upstream region. These observations suggest that the polyadenylation mechanisms behind each of the PAS variants may be considerably different. With these points in mind, we proposed a new set of features along with Omni-PolyA, a novel model for PAS prediction implemented as an online tool. To derive a robust model for each of the PAS variants, Omni-PolyA consists of a set of different classification models organized in a tree-like structure. To evaluate the performance of our model, we derived an Omni-PolyA model by using the DPS feature set proposed in [31], showing that Omni-PolyA consistently outperformed reported results by DPS (by 26.85%, Table 1). Next, we showed the performance of the model using the novel Omni-PolyA feature set, which reduced the average error rate by 35.37 and 41.34% compared to DPS and HMM_SVM state-of-the-art tools, respectively. Notably, the prediction of PAS-strong variants showed the most significant improvement, reducing the error rate by 36.95% and 49.50% compared to DPS and HMM_SVM, respectively. Finally, we used the GENCODE annotation (release 19) to obtain the recent curated human poly(A) data for GRCh37. Results in Table 3 show that Omni-PolyA consistently reduced the weighted average error rate by 6.86% and 20.63% compared to DPS and HMM_SVM, with the largest error reduction for the PAS-weak variants (11.50% and 32.64% relative to DPS and HMM_SVM tools, respectively).

## Methods

### Datasets

We considered two different datasets to assess the performance of the Omni-PolyA method. A PAS sequence is considered to be a genomic DNA sequence of 206 nt in length (100 nt downstream and 100 nt upstream flanking a PAS hexamer). The first dataset, proposed by Kalkatawi et al. [31, 32], is considered as a benchmark to compare against the state-of-the-art methods. This dataset contains 14,470 PAS-like sequences (7370 sequences with true PAS motif and 7370 pseudo-PAS sequences). The pseudo-PAS sequences contain canonical PAS hexamers (from the 12 PAS variants we considered) but with no links to the polyadenylation process. For each PAS variant, the number of sequences with true PAS hexamer and pseudo-PAS hexamer is selected to be the same. The true PAS sequences were obtained by mapping human mRNA sequences to the human genome (hg19). The pseudo-PAS sequences were randomly selected from human chromosome 21 after excluding the true PAS sequences.

The second dataset considered is based on the experimentally validated GENCODE annotation and is extracted from the human genome (hg19). We used the GENCODE PolyA feature annotation Release 19 (GRCh37.p13) [44], which contains polyA features manually annotated by the HAVANA group (http://www.sanger.ac.uk/science/groups/vertebrate-annotation). We used the information in the annotation file (GTF) to extract true PAS from the genome. In total, 18,786 sequences with true PAS were extracted for the 12 most frequent PAS variants in human. For each PAS variant, the same number of pseudo-PAS sequences was generated from human chromosome 21 after excluding all the true PAS sequences contained in that chromosome.

### Data normalization and cross-validation splits

**Fig. 6:**
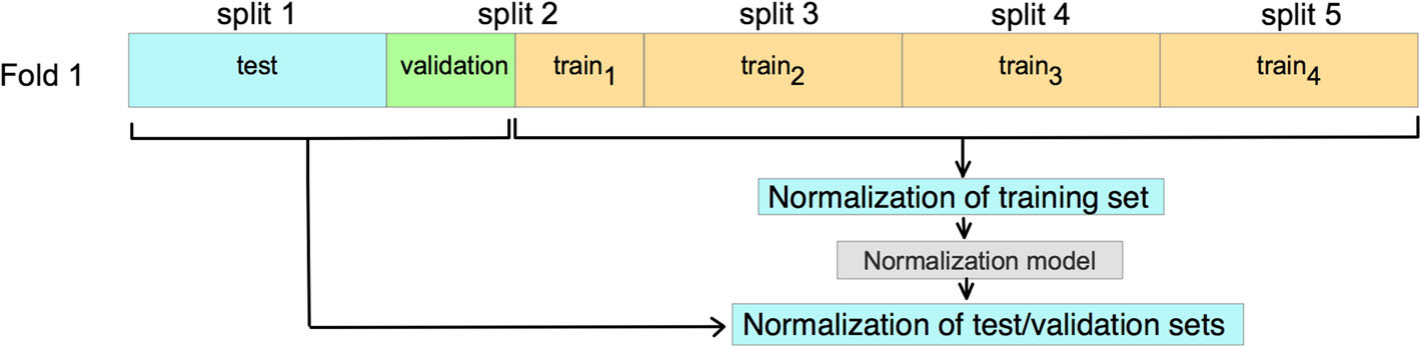
Data normalization procedure. Schematic representation of data normalization for fold 1 in a 5-fold cross-validation

We used the 5-fold cross-validation technique to validate the performance of all considered models. In the *k*-fold cross-validation, the original data is partitioned into *k* (approximately) equal-sized subsets. For each of the cross-validation folds, one of the subsets is used for testing the model while the remaining k-1 subsets are used to derive the classification model. Moreover, we reserved 15% of the training set for each fold as a validation set to optimize Omni-PolyA, DNN, DPS and, HMM_SVM model parameters. Therefore, the test set is exclusively used to assess the model performance in the final testing phase. Finally, feature values for all PAS variants were normalized to have values within the range of (−1, 1) according to

$$ {norm}_i=\frac{x_i-\left(\mathrm{ma}{\mathrm{x}}_i+\mathrm{mi}{\mathrm{n}}_i\right)/2}{\left(\mathrm{ma}{\mathrm{x}}_i-\mathrm{mi}{\mathrm{n}}_i\right)/2} $$

where *max* and *min* refer to the maximum and minimum values for *i*-th feature and *x* is the feature value that will be normalized. To avoid biased predictions, it is important to note that *max* and *min* values are obtained exclusively from the training data and are used as part of the model for the normalization of validation and test data (Fig. 6).

### Model training and data configuration

#### DPS model

We used DPS feature set to derive a random forest model as specified by Kalkatawi et al. [31]. Model parameters (i.e., the number of trees and number of randomly selected features) were determined based on the validation set (see Additional file 7: Table S4 for model parameters for each PAS variant).

#### HMM_SVM model

We derived HMM_SVM models by using the code provided by Xie et al. [33]. We optimized the model parameters (number of observations to combine into mega-state and the number of singular vectors to keep) by using a grid search method as specified by authors (see Additional file 7: Table S4 for model parameters for each PAS variant).

#### DNN model

We used MATLAB and Neural Network Toolbox release 2016a to derive DNN models with two autoencoder layers and one softmax layer. The number of units in the autoencoder layers was experimentally found by optimizing the error rate based on the validation set (see Additional file 7: Table S4 for model parameters for each PAS variant). DNN results in Table 1 and Table 3 show the performance of the models derived by using the DPS feature set (274 numeric features) [31].

#### Omni-PolyA model

**Fig. 7:**
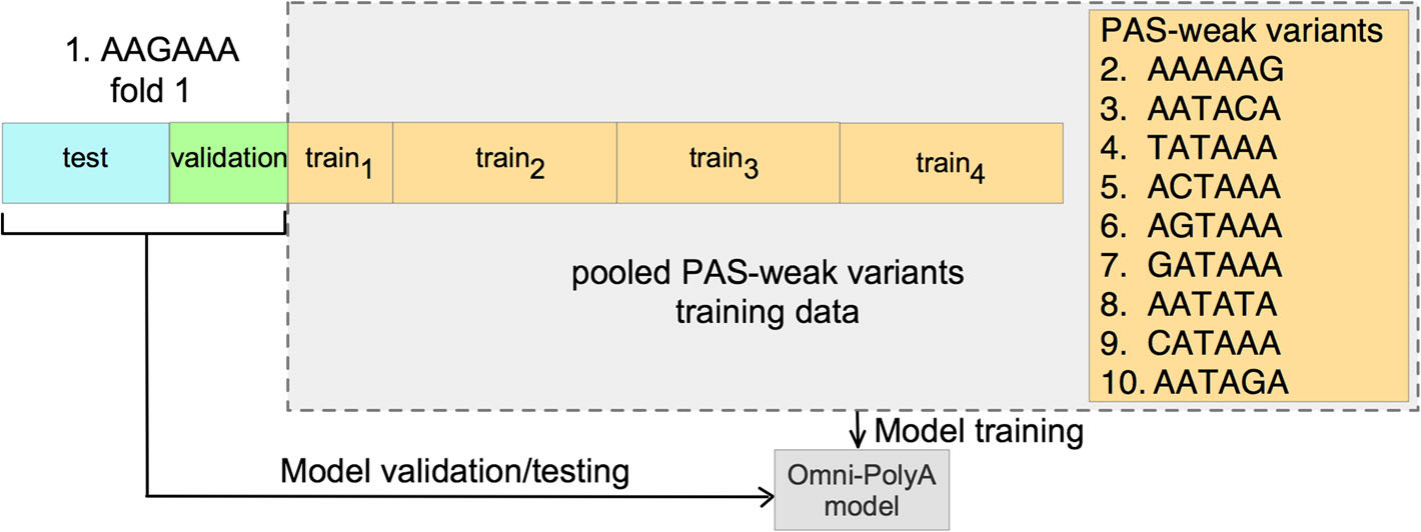
Schematic representation of Omni-PolyA model derived from pooled PAS-weak variants. The illustration shows the first fold of a 5-fold cross-validation technique for variant AAGAAA

Omni-PolyA uses four different classification models, namely, artificial neural network, random forest, C4.5 decision tree, and MLR models from WEKA v3.6.12 [61] and it is available as a MATLAB toolbox and as an online tool accessible at www.cbrc.kaust.edu.sa/omnipolya/. Moreover, columns sixth and seventh from Table 1 show the results obtained by the Omni-PolyA model derived by using the Omni-PolyA feature set (218 numeric features, listed in S1 Table). Finally, we pooled all PAS variants from the PAS-weak category to expand the training data. Therefore, the Omni-PolyA model was trained by using data from 10 PAS-weak variants and tested on the separate test set for a given variant (Fig. 7). Additional file 8: Table S5 shows the Omni-PolyA model parameters (determined from the validation set) for each PAS variant.

### Measures for assessing the model performance

As a representative measure of model performance, we used the classification error rate defined as

$$ errorrate= 1\hbox{-} \frac{TP+ TN}{TP+ TN+ FN+ FP}, $$

where TP, TN, FP and FN stand for the number of true positive predictions, true negative predictions, false positive predictions, and false negative predictions, respectively.

### Model-derived features

Here we show a brief description of features that are derived from a model using a portion of the training data. These features were inspired by those used by Magana-Mora et al. [62, 63] in models aimed to recognize translation initiation sites in plants genomic DNA.

#### PAS-score and pseudo PAS-score from 2-mer weight matrices

Using frequencies of the 16 di-nucleotide combinations (AA, AT, AC, AG, etc.), we derived two 2-mer weight matrices from the training data to represent the characteristics of both PAS and pseudo-PAS sequences separately. Both 2-mer weight matrices are then used to calculate a score (see below) indicating the likelihood of the sequence to be a functional PAS and a pseudo-PAS. Consequently, a PAS score and pseudo-PAS score is computed for each DNA sequence in the dataset and are calculated as follows: let S(a_j_) be a DNA sequence of length L, and P(p_ij_) be a 2-mer weight matrix of L-1 columns and 16 rows (for each di-nucleotide combination). The PAS score and pseudo-PAS score are given by

$$ \left[ PAS| pseudoPAS\right] score={\sum}_{i=1}^{16}{\sum}_{j=1}^{L-1}\mathrm{lo}{\mathrm{g}}_2\left(\frac{p_{ij}\times {a}_j{a}_{j+1}}{Pb_i}\right) $$

and

$$ {p}_{ij}\times {a}_j{a}_{j+1}=\left\{\begin{array}{cc}\hfill {p}_{ij},\hfill & \hfill {a}_j{a}_{j+1}={r}_i\hfill \\ {}\hfill 1,\hfill & \hfill {a}_j{a}_{j+1}\ne {r}_i\hfill \end{array}\right., $$

where *Pb*_*i*_ refers to the background probability from a uniform distribution.

#### Positional information gain score

We calculated the information gain independently for each position of the genomic sequence surrounding PAS. For this, we first computed the entropy of each position as follows: for a given position P in a training sequence we calculate the entropy for a nucleotide X as:

$$ E\left(P,X\right)=-\frac{c_1}{c_1+{c}_2}\mathrm{lo}{\mathrm{g}}_2\frac{c_1}{c_1+{c}_2}-\frac{c_2}{c_2+{c}_1}\log \frac{c_2}{c_2+{c}_1}, $$

where *c*_*1*_ represents the number of occurrences of nucleotide *X* (A, C, T or G) at position *P* in PAS sequences and *c*_*2*_ represents the number occurrences of the same nucleotide at position *P* in pseudo-PAS sequences. We also introduce another entropy measure at position *P* that adjusts for the proportion of PAS and pseudo-PAS samples in the training set in the following way

$$ E(P)=-\frac{c_1}{c_1+{c}_2}\mathrm{lo}{\mathrm{g}}_2\frac{c_1}{c_1+{c}_2}-\frac{c_2}{c_2+{c}_1}\log \frac{c_2}{c_2+{c}_1}, $$

where *c*_*1*_ and *c*_*2*_ indicate the number of PAS and pseudo PAS sequences in the training set, respectively. Finally, we calculated the information gain for a position *P* as defined in Russel and Norvig [64]:

$$ Gain(P)=E(P)-E\left(A,P\right)-E\left(C,P\right)-E\left(G,P\right)-E\left(T,P\right), $$

where A, C, G, and T refer to the four nucleotides. Finally, the sum of information gain for each position in the entire sequence (information gain score) is then used as one single feature. Therefore, samples with high and low information gain scores suggest PAS or pseudo-PAS sequences, respectively.

#### Nucleotide frequency of the most discriminative positions

We used the positional information gain (described above) for selecting the 40 most discriminant positions (20 from the upstream and 20 for the downstream regions relative to the PAS). We then counted the frequency of A, C, G, and T nucleotides in the 20 selected positions in the downstream and upstream, separately. Consequently, this results in eight numeric features denoting the frequency of A, C, G, and T in the most discriminant downstream and upstream positions.

## Additional files

Additional file 1: Table S1. Omni-PolyA feature set. List of the 218 numerical features. (PDF 104 kb)

Additional file 2: Table S2. Comparison of performances achieved by DPS, HMM_SVM, DNN, and Omni-PolyA. (PDF 110 kb)

Additional file 3: Table S3. False positive and false negative rates comparison between DPS, DNN, and Omni-polyA derived by using different feature sets. (PDF 105 kb)

Additional file 4: Figure S1. Nucleotide distribution for PAS variants in the PAS-weak category. These plots show the frequency of nucleotides for true PAS sequences in the 10 variants from the PAS-weak category. (PDF 1696 kb)

Additional file 5: Figure S2. DNA structural profiles of the PAS-strong variants. These plots represent the 16 considered structural profiles. Each structural profile is the average over all sequences from the PAS-strong variants (AATAAA and ATTAAA). These plots show the actual average values (y axis) over all sequences for each position (x axis). (PDF 2541 kb)

Additional file 6: Figure S3. DNA structural profiles of the PAS-weak variants. Each structural profile is the average over all sequences from the PAS-weak variants (10 PAS variants). These plots show the actual average values (y axis) over all sequences for each position (x axis). (PDF 2412 kb)

Additional file 7: Table S4. DPS, HMM_SVM and DNN model parameters. Parameters were determined from the validation set. (PDF 87 kb)

Additional file 8: Table S5. Omni-PolyA model parameters. Genetic algorithm parameters and feature set configuration determined from the validation set. (PDF 89 kb)

## Abbreviations

DNN: Deep neural networks
DPS: Dragon PolyA Spotter tool
FN: False negatives
FP: False positives
HMM_SVM: Tool based on hidden Markov model and support vector machine for poly(A) prediction
Nt: Nucleotides
PAS: Polyadenylation signal
SVM: Support vector machine
TN: True negatives
TP: True positives
