# Supplementary material for: Omni-PolyA: a method and tool for accurate recognition of Poly(A) signals in human genomic DNA
Source: BMC Genomics. 2017 Aug 15;18:620. doi: 10.1186/s12864-017-4033-7 (PMC5558757; doi:10.1186/s12864-017-4033-7)
Supplement: Supplementary file 1 — Omni-PolyA feature set. List of the 218 numerical features. (PDF 104 kb) [file 12864_2017_4033_MOESM1_ESM.pdf]

OMNI-POLYA: A METHOD AND TOOL FOR ACCURATE RECOGNITION OF POLY(A) SIGNALS IN HUMAN GENOMIC DNA

Arturo Magana-Mora[1], Manal Kalkatawi[1] and Vladimir B. Bajic[1,*]

[1]Computational Bioscience Research Center, King Abdullah University of Science and Technology (KAUST), Thuwal 23955-6900, Saudi Arabia.

* Corresponding author
E-mail: vladimir.bajic@kaust.edu.sa (VBB)

Table S1. Omni-PolyA feature set.

Contrast features - PAS vs pseudo-PAS	PAS score from 2-mer WMs
	pseudo-PAS score from 2-mer WMs
	A count 20 upstream positions (top ranked based on information gain)
	C count 20 upstream positions (top ranked based on information gain)
	G count 20 upstream positions (top ranked based on information gain)
	T count 20 upstream positions (top ranked based on information gain)
	A count 20 downstream positions (top ranked based on information gain)
	C count 20 downstream positions (top ranked based on information gain)
	G count 20 downstream positions (top ranked based on information gain)
	T count 20 downstream positions (top ranked based on information gain)
	Positional information gain score (average of the scores for each position)
RNA secondary structures	tetraloop ANYA=("ttgt" "ttat" "tagt" "taat" "tggt" "tgat" "tcgt" "tcat")
	tetraloop CUYG=("gagc" "gaac")
	tetraloop GNRA=("cttt" "ctct" "catt" "cact" "cgtt" "cgct" "cctt" "ccct")
	tetraloop UNCG=("atgc" "aagc" "aggc" "acgc")
	tetraloop UMAC=("attg" "agtg")
Nucleotide frequencies - codon position	Number of occurrences of nucleotide A in first position of upstream in-frame codons
	Number of occurrences of nucleotide A in second position of upstream in-frame codons
	Number of occurrences of nucleotide A in third position of upstream in-frame codons
	Number of occurrences of nucleotide C in first position of upstream in-frame codons
	Number of occurrences of nucleotide C in second position of upstream in-frame codons
	Number of occurrences of nucleotide C in third position of upstream in-frame codons
	Number of occurrences of nucleotide G in first position of upstream in-frame codons

	Number of occurrences of nucleotide G in second position of upstream in-frame codons
	Number of occurrences of nucleotide G in third position of upstream in-frame codons
	Number of occurrences of nucleotide T in first position of upstream in-frame codons
	Number of occurrences of nucleotide T in second position of upstream in-frame codons
	Number of occurrences of nucleotide T in third position of upstream in-frame codons
	Number of occurrences of nucleotide A in first position of downstream in-frame codons
	Number of occurrences of nucleotide A in second position of downstream in-frame codons
	Number of occurrences of nucleotide A in third position of downstream in-frame codons
	Number of occurrences of nucleotide C in first position of downstream in-frame codons
	Number of occurrences of nucleotide C in second position of downstream in-frame codons
	Number of occurrences of nucleotide C in third position of downstream in-frame codons
	Number of occurrences of nucleotide G in first position of downstream in-frame codons
	Number of occurrences of nucleotide G in second position of downstream in-frame codons
	Number of occurrences of nucleotide G in third position of downstream in-frame codons
	Number of occurrences of nucleotide T in first position of downstream in-frame codons
	Number of occurrences of nucleotide T in second position of downstream in-frame codons
	Number of occurrences of nucleotide T in third position of downstream in-frame codons
1-mer frequencies	1-mer upstream: A
	1-mer upstream: C
	1-mer upstream: G
	1-mer upstream: T
	1-mer downstream: A
	1-mer downstream: C
	1-mer downstream: G
	1-mer downstream: T
2-mer	2-mer: upstream: AA

fequencies	2-mer: upstream: AC
	2-mer: upstream: AG
	2-mer: upstream: AT
	2-mer: upstream: CA
	2-mer: upstream: CC
	2-mer: upstream: CG
	2-mer: upstream: CT
	2-mer: upstream: GA
	2-mer: upstream: GC
	2-mer: upstream: GG
	2-mer: upstream: GT
	2-mer: upstream: TA
	2-mer: upstream: TC
	2-mer: upstream: TG
	2-mer: upstream: TT
	2-mer: downstream: AA
	2-mer: downstream: AC
	2-mer: downstream: AG
	2-mer: downstream: AT
	2-mer: downstream: CA
	2-mer: downstream: CC
	2-mer: downstream: CG
	2-mer: downstream: CT
	2-mer: downstream: GA
	2-mer: downstream: GC
	2-mer: downstream: GG
	2-mer: downstream: GT
	2-mer: downstream: TA
	2-mer: downstream: TC
	2-mer: downstream: TG
	2-mer: downstream: TT
structural profile - aphicility	average of region [-100,76]
	average of region [-75,-51]
	average of region [-50,-26]
	average of region [-25,-1]
	average of region [0,24]
	average of region [25,49]
	average of region [50,74]
	average of region [75,99]
structural profile - base	average of region [-100,76]
	average of region [-75,-51]

stacking	average of region [-50,-26]
	average of region [-25,-1]
	average of region [0,24]
	average of region [25,49]
	average of region [50,74]
	average of region [75,99]
structural profile - bdna twist	average of region [-100,76]
	average of region [-75,-51]
	average of region [-50,-26]
	average of region [-25,-1]
	average of region [0,24]
	average of region [25,49]
	average of region [50,74]
	average of region [75,99]
structural profile - bendability	average of region [-100,76]
	average of region [-75,-51]
	average of region [-50,-26]
	average of region [-25,-1]
	average of region [0,24]
	average of region [25,49]
	average of region [50,74]
	average of region [75,99]
structural profile - bending stiffness	average of region [-100,76]
	average of region [-75,-51]
	average of region [-50,-26]
	average of region [-25,-1]
	average of region [0,24]
	average of region [25,49]
	average of region [50,74]
	average of region [75,99]
structural profile - cpgislands	average of region [-100,76]
	average of region [-75,-51]
	average of region [-50,-26]
	average of region [-25,-1]
	average of region [0,24]
	average of region [25,49]
	average of region [50,74]
	average of region [75,99]
structural profile - cpnpcpgislans	average of region [-100,76]
	average of region [-75,-51]
	average of region [-50,-26]

	average of region [-25,-1]
	average of region [0,24]
	average of region [25,49]
	average of region [50,74]
	average of region [75,99]
structural profile - cpnpg islands	average of region [-100,76]
	average of region [-75,-51]
	average of region [-50,-26]
	average of region [-25,-1]
	average of region [0,24]
	average of region [25,49]
	average of region [50,74]
	average of region [75,99]
structural profile - dna denaturation	average of region [-100,76]
	average of region [-75,-51]
	average of region [-50,-26]
	average of region [-25,-1]
	average of region [0,24]
	average of region [25,49]
	average of region [50,74]
	average of region [75,99]
structural profile - duplex stability disrupt energy	average of region [-100,76]
	average of region [-75,-51]
	average of region [-50,-26]
	average of region [-25,-1]
	average of region [0,24]
	average of region [25,49]
	average of region [50,74]
	average of region [75,99]
structural profile - duplex stability free energy	average of region [-100,76]
	average of region [-75,-51]
	average of region [-50,-26]
	average of region [-25,-1]
	average of region [0,24]
	average of region [25,49]
	average of region [50,74]
	average of region [75,99]
structural profile - nucleosome position	average of region [-100,76]
	average of region [-75,-51]
	average of region [-50,-26]
	average of region [-25,-1]

	average of region [0,24]
	average of region [25,49]
	average of region [50,74]
	average of region [75,99]
structural profile - propellor twist	average of region [-100,76]
	average of region [-75,-51]
	average of region [-50,-26]
	average of region [-25,-1]
	average of region [0,24]
	average of region [25,49]
	average of region [50,74]
	average of region [75,99]
structural profile - protein deformation	average of region [-100,76]
	average of region [-75,-51]
	average of region [-50,-26]
	average of region [-25,-1]
	average of region [0,24]
	average of region [25,49]
	average of region [50,74]
	average of region [75,99]
structural profile - protein dna twist	average of region [-100,76]
	average of region [-75,-51]
	average of region [-50,-26]
	average of region [-25,-1]
	average of region [0,24]
	average of region [25,49]
	average of region [50,74]
	average of region [75,99]
structural profile - zdna	average of region [-100,76]
	average of region [-75,-51]
	average of region [-50,-26]
	average of region [-25,-1]
	average of region [0,24]
	average of region [25,49]
	average of region [50,74]
	average of region [75,99]
others	kozak
	number of ATG inframe downstream
	number of ATG outframe downstream
	number of C nucleotide in the upstream region
	number of ATG inframe upstream

	number of ATG outframe upstream
	total number of ATG codons in upstream+downstream
	kozak consensus
	Score (0-6) # of A in positions 174, 177, 180, 183, 186, 189
	binary, in-frame stop codon (tag, taa, tga)
