# Supplementary material for: Omni-PolyA: a method and tool for accurate recognition of Poly(A) signals in human genomic DNA
Source: BMC Genomics. 2017 Aug 15;18:620. doi: 10.1186/s12864-017-4033-7 (PMC5558757; doi:10.1186/s12864-017-4033-7)
Supplement: Supplementary file 2 — Comparison of performances achieved by DPS, HMM_SVM, DNN, and Omni-PolyA. (PDF 110 kb) [file 12864_2017_4033_MOESM2_ESM.pdf]

OMNI-POLYA: A METHOD AND TOOL FOR ACCURATE RECOGNITION OF POLY(A) SIGNALS IN HUMAN GENOMIC DNA

Arturo Magana-Mora[1], Manal Kalkatawi[1] and Vladimir B. Bajic[1,*]

[1]Computational Bioscience Research Center, King Abdullah University of Science and Technology (KAUST), Thuwal 23955-6900, Saudi Arabia.

* Corresponding author

E-mail: vladimir.bajic@kaust.edu.sa (VBB)

Table S2. Comparison of performances achieved by DPS, HMM_SVM, DNN and Omni-PolyA.

Variants	Size	Error rate (%)				False positive rate (%)				False negative rate (%)			
		DPS§	HMM_SVM	DNN§	Omni-PolyA§	DPS§	HMM_SVM	DNN§	Omni-PolyA§	DPS§	HMM_SVM	DNN§	Omni-PolyA§
AATAAA	5190	23.72	28.13	16.80	**14.02**	33.64	30.02	15.37	**11.41**	13.80	26.86	18.22	**16.65**
ATTAAA	2400	16.63	23.96	15.50	**14.00**	17.08	24.42	15.08	**12.50**	16.17	23.48	15.91	**15.50**
AAGAAA	1250	14.00	**10.96**	16.88	11.84	13.44	**8.48**	16.00	14.08	14.56	13.44	17.76	**9.60**
AAAAAG	1230	8.05	8.62	8.29	**4.87**	10.08	**2.93**	7.31	5.53	6.02	14.31	9.26	**4.23**
AATACA	880	20.00	19.89	17.72	**13.52**	21.14	**12.27**	19.09	13.18	18.86	27.50	16.36	**13.86**
TATAAA	780	18.08	**16.79**	21.28	20.38	16.15	**12.31**	19.74	25.13	20.00	21.28	22.82	**15.64**
ACTAAA	690	23.33	26.38	23.04	**19.56**	24.06	19.42	21.15	**18.55**	22.61	33.04	24.92	**20.58**
AGTAAA	670	19.55	23.13	22.98	**16.71**	17.01	**16.72**	23.28	17.31	22.09	29.55	22.68	**16.12**
GATAAA	460	21.74	**12.83**	16.73	13.69	27.39	**3.04**	17.82	13.48	16.09	22.61	15.65	**13.91**
AATATA	410	18.05	**14.15**	20.00	16.82	15.61	**4.39**	19.02	20.00	20.49	23.90	20.97	**13.66**
CATAAA	410	20.00	**14.15**	26.34	24.14	19.51	**5.37**	20.97	28.78	20.49	22.93	25.36	**19.51**
AATAGA	370	18.38	**8.11**	15.40	12.93	19.46	**3.78**	11.89	14.05	17.30	12.43	18.91	**11.89**
Average		19.25	21.21	17.07	**14.08**	22.94	19.02	16.03	**13.61**	15.55	23.59	17.92	**14.56**

'Size' corresponds to the number of samples for each motif variant. The *'error rate'* is the percentage of misclassified motifs, and it is equal to 1-accuracy. The *'false positive rate'* denotes the probability of incorrectly classifying a pseudo-PAS as PAS and it is equal to 1-specificity. The *'false negative rate'* stands for the probability of falsely classifying a PAS as a pseudo-PAS, and it is equal to 1-sensitivity. DPS and HMM_SVM results correspond to those reported by Kalkatawi *et al*. [1]and Xie *et al*. [2], respectively. Average denotes the weighted average of a column. The best performing model for each PAS variant is highlighted in bold. Symbol § indicates that models were derived using the same DPS feature set.

References

1. Kalkatawi M, Rangkuti F, Schramm M, Jankovic BR, Kamau A, Chowdary R, et al. Dragon PolyA Spotter: predictor of poly(A) motifs within human genomic DNA sequences. Bioinformatics. 2013;29:11:1484.
2. Xie B, Jankovic BR, Bajic VB, Song L, Gao X. Poly(A) motif prediction using spectral latent features from human DNA sequences. Bioinformatics. 2013;29:13:i316-i325.
