# Supplementary material for: Omni-PolyA: a method and tool for accurate recognition of Poly(A) signals in human genomic DNA
Source: BMC Genomics. 2017 Aug 15;18:620. doi: 10.1186/s12864-017-4033-7 (PMC5558757; doi:10.1186/s12864-017-4033-7)
Supplement: Supplementary file 3 — False positive and false negative rates comparison between DPS, DNN, and Omni-polyA derived by using different feature sets. (PDF 105 kb) [file 12864_2017_4033_MOESM3_ESM.pdf]

Omni-PolyA: A Method and Tool for Accurate Recognition of Poly(A) Signals in Human Genomic DNA

Arturo Magana-Mora[1], Manal Kalkatawi[1] and Vladimir B. Bajic[1,*]

[1]Computational Bioscience Research Center, King Abdullah University of Science and Technology (KAUST), Thuwal 23955-6900, Saudi Arabia.

* Corresponding author
E-mail: vladimir.bajic@kaust.edu.sa (VBB)

Table S3. False positive and false negative rates comparison between DPS, DNN, and Omni-PolyA derived using different feature sets.

Variants	Size	False positive rate (%)					False negative rate (%)				
		DPS model	DNN model	Omni-PolyA	Omni-PolyA	Omni-PolyA	DPS model	DNN model	Omni-PolyA	Omni-PolyA	Omni-PolyA
		DPS feature set	DPS feature set	DPS feature set	Omni-PolyA feature set	Omni-PolyA feature set PAS-weak data pooled	DPS feature set	DPS feature set	DPS feature set	Omni-PolyA feature set	Omni-PolyA feature set PAS-weak data pooled
AATAAA	5190	33.64	15.37	11.41	**13.60**	**13.60**	13.80	18.22	16.65	**14.80**	**14.80**
ATTAAA	2400	17.08	15.08	12.50	**9.25**	**9.25**	16.17	15.91	**15.50**	15.75	15.75
AAGAAA	1250	13.44	16.00	14.08	9.76	**7.52**	14.56	17.76	**9.60**	11.84	15.20
AAAAAG	1230	10.08	7.31	5.53	6.99	**6.67**	6.02	9.26	**4.23**	4.72	**4.23**
AATACA	880	21.14	19.09	13.18	**11.82**	12.05	18.86	16.36	**13.86**	16.36	15.00
TATAAA	780	16.15	19.74	25.13	15.64	**13.59**	20.00	22.82	15.64	**13.85**	14.10
ACTAAA	690	24.06	21.15	18.55	17.39	**10.14**	22.61	24.92	20.58	**15.07**	18.84
AGTAAA	670	17.01	23.28	17.31	11.64	**8.06**	22.09	22.68	**16.12**	17.91	18.21
GATAAA	460	27.39	17.82	13.48	11.74	**6.96**	16.09	15.65	13.91	9.57	**10.00**
AATATA	410	15.61	19.02	20.00	16.10	**12.20**	20.49	20.97	**13.66**	15.61	14.63
CATAAA	410	19.51	20.97	28.78	**11.22**	13.66	20.49	25.36	19.51	17.56	**15.12**
AATAGA	370	19.46	11.89	14.05	**13.51**	15.14	17.30	18.91	11.89	12.43	**8.11**
Average		22.94	16.03	13.61	12.05	**11.08**	15.55	17.92	14.56	**13.93**	14.11

'*Size*' corresponds to the number of samples for each motif variant. The *'false positive rate'* denotes the probability of incorrectly classifying a pseudo-PAS as PAS and it is equal to 1-specificity. The *'false negative rate'* stands for the probability of falsely classifying a PAS as a pseudo-PAS, and it is equal to 1-sensitivity. DPS results correspond to those reported by Kalkatawi *et al*. [1]. Average denotes the weighted average of a column. The best performing model for each PAS variant is highlighted in bold.

References

1. Kalkatawi M, Rangkuti F, Schramm M, Jankovic BR, Kamau A, Chowdary R, et al. Dragon PolyA Spotter: predictor of poly(A) motifs within human genomic DNA sequences. Bioinformatics. 2013;29:11:1484.
