# Supplementary material for: Omni-PolyA: a method and tool for accurate recognition of Poly(A) signals in human genomic DNA
Source: BMC Genomics. 2017 Aug 15;18:620. doi: 10.1186/s12864-017-4033-7 (PMC5558757; doi:10.1186/s12864-017-4033-7)

Omni-PolyA: A Method and Tool for Accurate Recognition of Poly(A) Signals in Human Genomic DNA

Arturo Magana-Mora[1], Manal Kalkatawi[1] and Vladimir B. Bajic[1,*]

[1]Computational Bioscience Research Center, King Abdullah University of Science and Technology (KAUST), Thuwal 23955-6900, Saudi Arabia.

* Corresponding author
E-mail: vladimir.bajic@kaust.edu.sa (VBB)

Figure S1. Nucleotide distribution for PAS variants in PAS-weak category. These plots show the frequency of the nucleotides for true PAS sequences in the 10 variants from PAS-weak category.

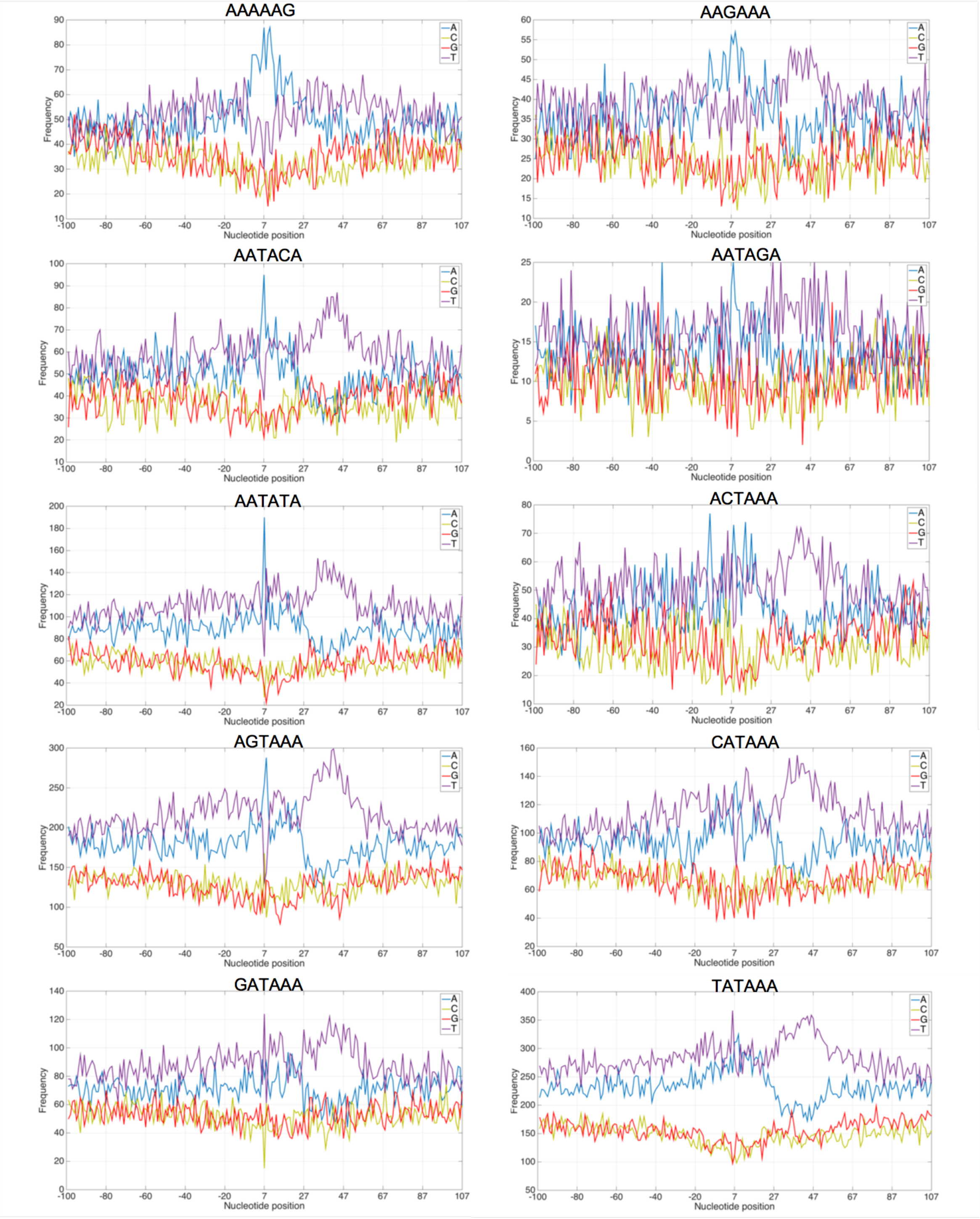

Supplement: Supplementary file 4 — Nucleotide distribution for PAS variants in the PAS-weak category. These plots show the frequency of nucleotides for true PAS sequences in the 10 variants from the PAS-weak category. (PDF 1696 kb) [file 12864_2017_4033_MOESM4_ESM.pdf]
