# Supplementary material for: Omni-PolyA: a method and tool for accurate recognition of Poly(A) signals in human genomic DNA
Source: BMC Genomics. 2017 Aug 15;18:620. doi: 10.1186/s12864-017-4033-7 (PMC5558757; doi:10.1186/s12864-017-4033-7)

Omni-PolyA: A Method and Tool for Accurate Recognition of Poly(A) Signals in Human Genomic DNA

Arturo Magana-Mora[1], Manal Kalkatawi[1] and Vladimir B. Bajic[1,*]

[1]Computational Bioscience Research Center, King Abdullah University of Science and Technology (KAUST), Thuwal 23955-6900, Saudi Arabia.

* Corresponding author
E-mail: vladimir.bajic@kaust.edu.sa (VBB)

Figure S2. DNA structural profiles of the PAS-strong variants. These plots represent the 16 considered structural profiles. Each structural profile is the average over all sequences from the PAS-strong variants (AATAAA and ATTAAA). These plots show the actual average values (y axis) over all sequences for each position (x axis).

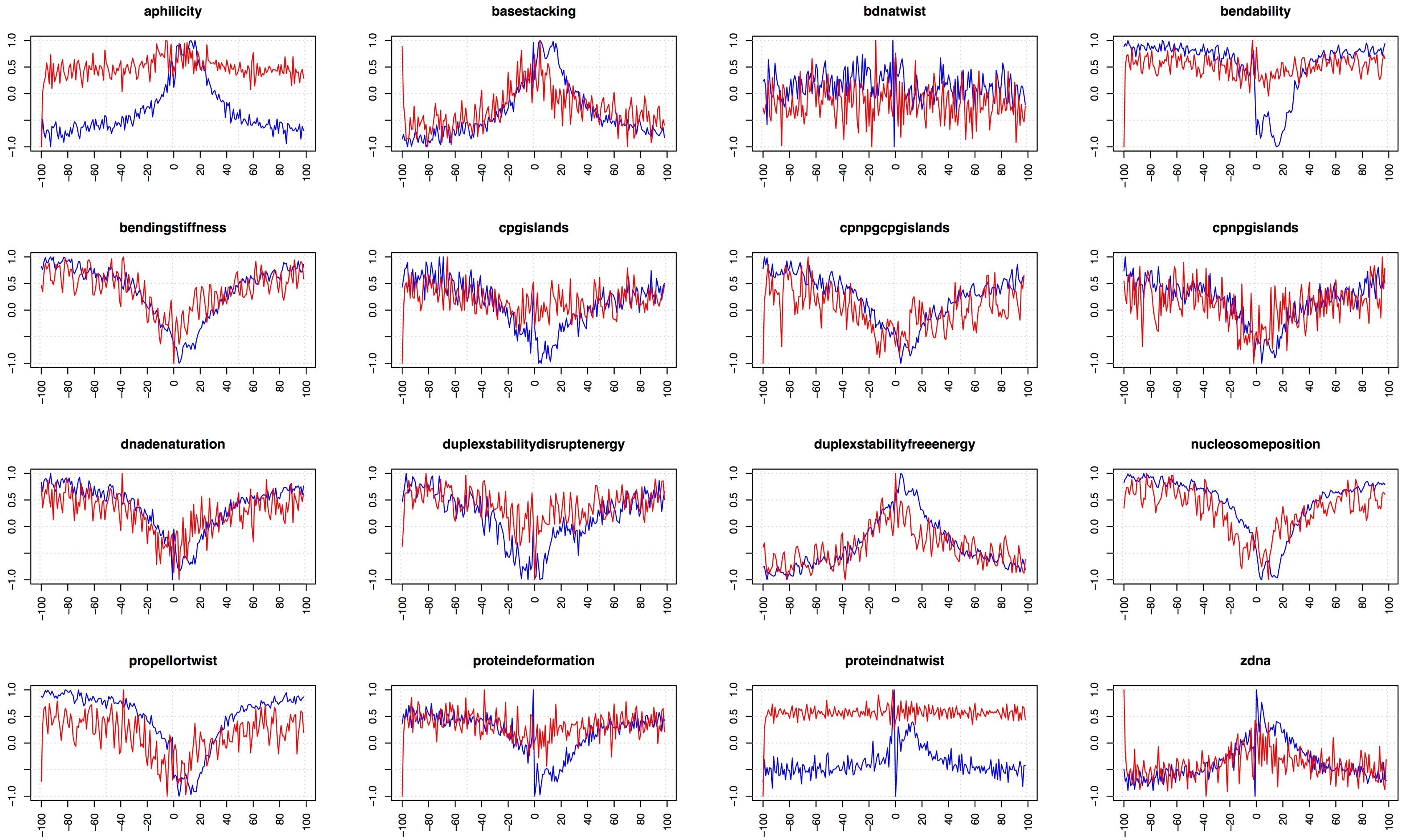

Supplement: Supplementary file 5 — DNA structural profiles of the PAS-strong variants. These plots represent the 16 considered structural profiles. Each structural profile is the average over all sequences from the PAS-strong variants (AATAAA and ATTAAA). These plots show the actual average values (y axis) over all sequences for each position (x axis). (PDF 2541 kb) [file 12864_2017_4033_MOESM5_ESM.pdf]
