# Supplementary material for: Omni-PolyA: a method and tool for accurate recognition of Poly(A) signals in human genomic DNA
Source: BMC Genomics. 2017 Aug 15;18:620. doi: 10.1186/s12864-017-4033-7 (PMC5558757; doi:10.1186/s12864-017-4033-7)

OMNI-POLYA: A METHOD AND TOOL FOR ACCURATE RECOGNITION OF POLY(A) SIGNALS IN HUMAN GENOMIC DNA

Arturo Magana-Mora[1], Manal Kalkatawi[1] and Vladimir B. Bajic[1,*]

[1]Computational Bioscience Research Center, King Abdullah University of Science and Technology (KAUST), Thuwal 23955-6900, Saudi Arabia.

* Corresponding author
E-mail: vladimir.bajic@kaust.edu.sa (VBB)

Figure S3. DNA structural profiles of the PAS-weak variants. Each structural profile is the average over all sequences from the PAS-weak variants (10 PAS variants). These plots show the actual average values (y axis) over all sequences for each position (x axis).

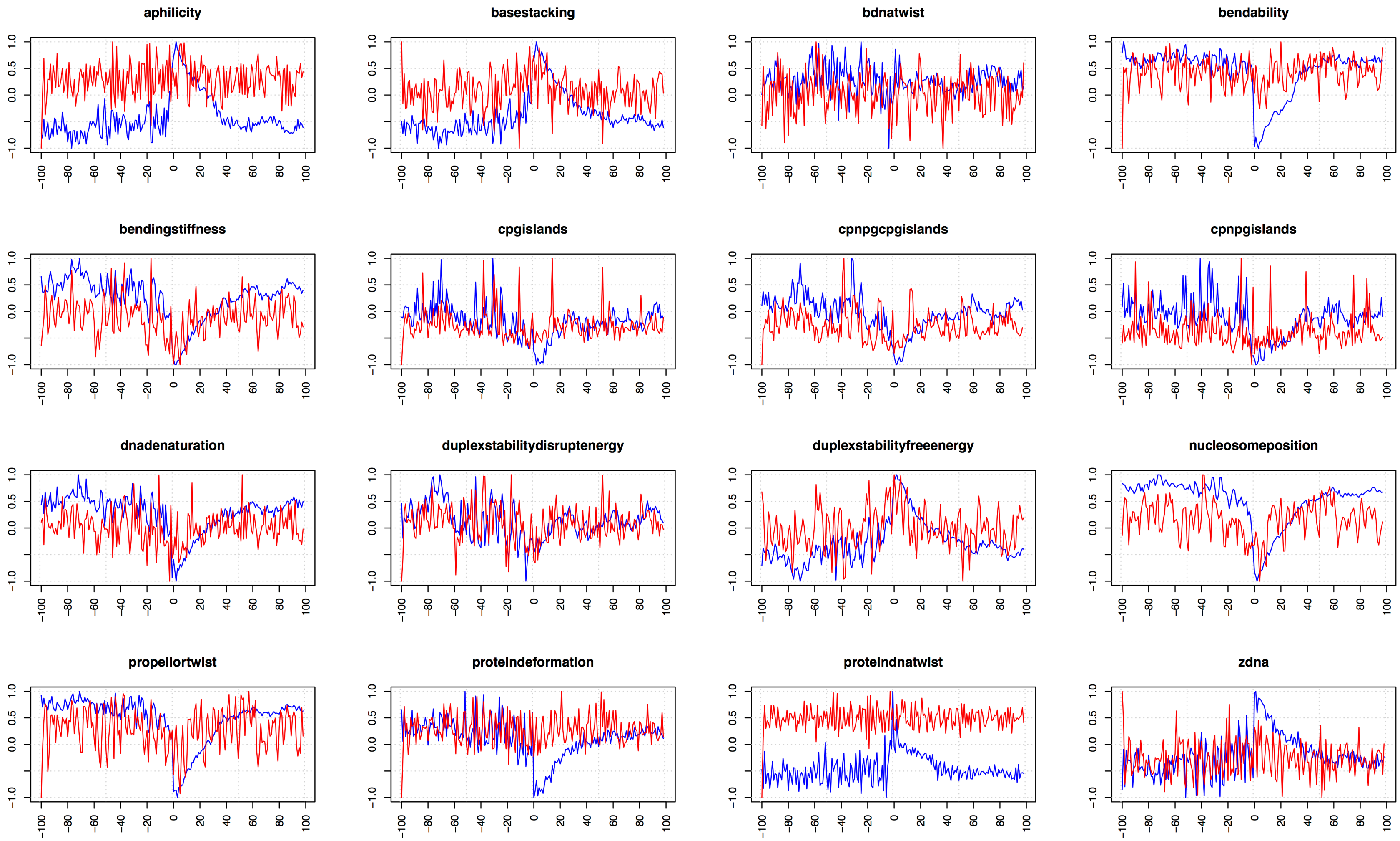

Supplement: Supplementary file 6 — DNA structural profiles of the PAS-weak variants. Each structural profile is the average over all sequences from the PAS-weak variants (10 PAS variants). These plots show the actual average values (y axis) over all sequences for each position (x axis). (PDF 2412 kb) [file 12864_2017_4033_MOESM6_ESM.pdf]
