# Supplementary material for: Omni-PolyA: a method and tool for accurate recognition of Poly(A) signals in human genomic DNA
Source: BMC Genomics. 2017 Aug 15;18:620. doi: 10.1186/s12864-017-4033-7 (PMC5558757; doi:10.1186/s12864-017-4033-7)
Supplement: Supplementary file 7 — DPS, HMM_SVM and DNN model parameters. Parameters were determined from the validation set. (PDF 87 kb) [file 12864_2017_4033_MOESM7_ESM.pdf]

OMNI-POLYA: A METHOD AND TOOL FOR ACCURATE RECOGNITION OF POLY(A) SIGNALS IN HUMAN GENOMIC DNA

Arturo Magana-Mora[1], Manal Kalkatawi[1] and Vladimir B. Bajic[1,*]

[1]Computational Bioscience Research Center, King Abdullah University of Science and Technology (KAUST), Thuwal 23955-6900, Saudi Arabia.

* Corresponding author
E-mail: vladimir.bajic@kaust.edu.sa (VBB)

Table S4. DPS, HMM_SVM and DNN models parameters. Parameters were determined from the validation set.

Variants	DPS parameters		HMM_SVM parameters		DNN parameters	
	Number of trees	Number of features	Number latent var.	Number of observations	Number units layer 1	Number units layer 2
AATAAA	120	12	40	6	145	45
ATTAAA	90	12	2	4	150	10
AAGAAA	100	12	38	6	165	10
AAAAAG	120	15	4	7	75	35
AATACA	20	6	36	7	60	60
TATAAA	120	15	4	5	195	50
ACTAAA	20	15	2	7	145	45
AGTAAA	120	12	20	5	170	60
GATAAA	10	15	34	7	130	80
AATATA	110	12	2	6	100	35
CATAAA	80	15	4	6	110	70
AATAGA	110	9	2	6	105	35

'Number of trees' and *'number of features'* represent the parameters for the RF model as described in [1]. *'Number of latent var.'* and *'Number of observations'* refer to the parameters for the HMM_SVM model [2] *'Number units layer n'* corresponds to the number of autoencoder units used for each of the hidden layers. The parameters for both autoencoders are: max epochs = 500 (layer 1) and 200 (layer 2), L2WeightRegularization = 0.004 (layer 1) and 0.002 (layer 2), and SparsityRegularization = 4 (for both layers). Finally, the number of max epoch for the softlayer was set to 400. In DPS, HMM_SVM and DNN models, parameters were found by using a grid search based on the validation set.

References

1. Kalkatawi M, Rangkuti F, Schramm M, Jankovic BR, Kamau A, Chowdary R, et al. Dragon PolyA Spotter: predictor of poly(A) motifs within human genomic DNA sequences. Bioinformatics. 2013;29:11:1484.
2. Xie B, Jankovic BR, Bajic VB, Song L, Gao X. Poly(A) motif prediction using spectral latent features from human DNA sequences. Bioinformatics. 2013;29:13:i316-i325.
