# Supplementary material for: Omni-PolyA: a method and tool for accurate recognition of Poly(A) signals in human genomic DNA
Source: BMC Genomics. 2017 Aug 15;18:620. doi: 10.1186/s12864-017-4033-7 (PMC5558757; doi:10.1186/s12864-017-4033-7)
Supplement: Supplementary file 8 — Omni-PolyA model parameters. Genetic algorithm parameters and feature set configuration determined from the validation set. (PDF 89 kb) [file 12864_2017_4033_MOESM8_ESM.pdf]

OMNI-POLYA: A METHOD AND TOOL FOR ACCURATE RECOGNITION OF POLY(A) SIGNALS IN HUMAN GENOMIC DNA

Arturo Magana-Mora[1], Manal Kalkatawi[1] and Vladimir B. Bajic[1,*]

[1]Computational Bioscience Research Center, King Abdullah University of Science and Technology (KAUST), Thuwal 23955-6900, Saudi Arabia.

* Corresponding author
E-mail: vladimir.bajic@kaust.edu.sa (VBB)

Table S5. Omni-PolyA model parameters.

Variants	Omni-PolyA Parameters				
	GA # iterations	GA size population	Crossover probability	Mutation probability	Feature set
AATAAA	15	30	0.90	0.20	DPS
ATTAAA	15	30	0.55	0.15	OPS
AAGAAA	15	30	0.95	0.25	OPS
AAAAAG	15	30	0.85	0.25	DPS
AATACA	15	30	0.85	0.05	DPS
TATAAA	15	30	0.55	0.15	OPS + pooling
ACTAAA	15	30	0.85	0.05	OPS + pooling
AGTAAA	15	30	0.70	0.10	OPS + pooling
GATAAA	15	30	0.95	0.05	OPS + pooling
AATATA	15	30	0.90	0.10	OPS + pooling
CATAAA	15	30	0.85	0.05	OPS + pooling
AATAGA	15	30	0.95	0.10	OPS + pooling

'*Feature set*' indicates the dataset used to derived the Omni-PolyA model, where DPS and OPS stand for the feature set proposed in DragonPolyA [1, 2] and the feature set proposed in this study, respectively. Finally, '*pooling*' indicates that Omni-PolyA model was derived from pooled data from PAS-weak variants. GA parameters and feature set configurations were determined from minimizing the error rate from the validation set.

References

1. Kalkatawi M, Rangkuti F, Schramm M, Jankovic BR, Kamau A, Chowdary R, et al. Dragon PolyA Spotter: predictor of poly(A) motifs within human genomic DNA sequences. Bioinformatics. 2013;29:11:1484.
2. Kalkatawi M, Rangkuti F, Schramm M, Jankovic BR, Kamau A, Chowdary R, et al. Dragon PolyA Spotter: predictor of poly(A) motifs within human genomic DNA sequences. Bioinformatics. 2012;28:1:127-129.
